# Overcoming barriers to sustainable agricultural intensification

**DOI:** 10.1098/rsos.260775

**Published:** 2026-10-21

**Authors:** Rachael Garrett

**Affiliations:** Conservation and Development Lab, Conservation Research Institute and Department of Geography, University of Cambridge, Cambridge, UK

**Keywords:** food security, conservation, yield gaps, agricultural expansion, land sparing, tropics, Latin America, sub-Saharan Africa, socio-technical transition, behavioural change

## Abstract

Sustainably closing agricultural yield gaps remains a central challenge for advancing global food security while conserving biodiversity. This article develops a theoretical perspective on why sustainable intensification (SI) is limited in many lower-income countries, despite the technological feasibility of improving yields in these places. It explores these constraints to SI through two case studies of expanding commodity frontiers: (i) beef in the Brazilian Amazon and Cerrado and (ii) cocoa in West Africa. We find similar barriers to SI across the cases, including limited tenurial, financial, knowledge and cultural support, community-level risks to investment and cheap land favouring agricultural expansion. At the same time, both cases reveal potential for rapid intensification, as demonstrated by success in selected locations. The analysis helps explain why many promising SI strategies have achieved limited uptake in countries where land for expansion is still relatively abundant. The article also identifies a more optimistic pathway: ambitious economic and institutional reforms, well-funded community-level technical and financial investments, and smart conservation policies could unlock joint gains for food security and biodiversity. Global policy and finance changes have opened windows for this coordinated policy agenda, but improved demand-side policies are also needed to tip low-yielding agricultural systems onto new trajectories.

## 1. Introduction

In the last 50 years, the productivity of many major crops has increased steadily. Yet in many locations, and for some crops, yields have failed to keep pace with broader technological developments [1]. Across much of the world, large differences remain between the agronomically attainable productivity of crops and what is achieved in farmers’ fields. These **yield gaps** are particularly high in low- and middle-income countries, exacerbating other development and equity concerns [2]. Moreover, women and other vulnerable communities tend to suffer more from the conditions that contribute to yield gaps (e.g. insecure tenure and lower resource access) than more privileged farmers [3,4].

While increasing yields may involve trade-offs across different sustainability goals [5,6], there is substantial scope to increase production in ways that deliver high social benefits and low environmental harm [7]. Crucially, regions with the largest yield gaps often overlap with areas of globally important yet highly threatened biodiversity. There are many low- to middle-income countries in the tropics that have exceptional species endemism and richness, high carbon stocks [8] and some of the world's largest yield gaps, e.g. Brazil, the Democratic Republic of Congo, Ethiopia, Ghana, Guatemala, India, Indonesia, Kenya, Nigeria and Venezuela, among others.

Low productivity requires more land to produce the same amount of food. Moreover, in the tropics, productivity in smallholder agricultural systems often falls rapidly over time owing to nutrient depletion and soil degradation. When this happens, farmers tend to seek out new agricultural areas, most often at the expense of intact or recovering natural ecosystems. Indeed, many of these same regions with high agricultural expansion into natural ecosystems [9] are the same as those with high yield gaps [2,10], including Ghana, Brazil and Indonesia. Closing these yield gaps is therefore a central concern for achieving food security and resilient livelihoods without exacerbating the ongoing biodiversity and climate crises.

Efforts to increase agricultural output from existing land while maintaining or enhancing environmental integrity and resilience are commonly described as **sustainable intensification** (SI) [11]. A wide range of agronomic and ecological approaches have demonstrated the capacity to increase yields with relatively low environmental cost. These include improved cultivars, more efficient and precise fertilizer use that reduces nutrient losses, crop rotations, crop–livestock integration, green manuring systems, reduced tillage, water-saving irrigation technologies and certain agroforestry approaches, among others [12–14].

Despite the potential benefits of SI and the range of technological and management changes available to boost yields, the adoption of such practices remains limited in many regions. This raises a fundamental question: *if SI practices can deliver so many socio-economic and environmental benefits, why are they not more widely adopted?*

This article explores this question through two case studies exemplifying the overlap of large yield gaps and highly threatened biodiversity: beef cattle pastures in the Brazilian Amazon and Cerrado ecosystems and cocoa production systems in the Upper Guinean Forests (UGF) of West Africa. Before discussing these cases, we present several interdisciplinary structural and behavioural theories that can help illuminate the complex causes of existing agricultural practices and the theoretical conditions under which SI can occur.

## 2. Conceptual framework

### 2.1. A behavioural perspective

Many conventional economic models assume that land users exhibit ‘rational’ decision-making. Under this perspective, a farmer is expected to adopt a technology if the overall utility (expected net well-being benefit) of adopting the technology over some future period (considering the probabilities of different cost and benefit flows) is higher than the utility of not adopting. Conceptualizations of utility are often focused on expected financial returns (i.e. profit maximization), but can also include non-financial aspects like labour and leisure trade-offs. Since most technologies that improve yields (e.g. fertilizer applications) face diminishing benefits relative to costs at higher levels of adoption, there are very often differences between the yield-maximizing and profit-maximizing levels of adoption. The literature also focuses on constraints to adoption even where a certain adoption level is otherwise profit-maximizing, including affordability and access, as well as awareness (education and learning processes), which are in turn related to local economies of scale, credit and capital constraints and risk [15]. Numerous case studies about agricultural technologies in low-income regions, some of which can be considered SI practices, further document how the failings of input markets, limitations in knowledge and education, and problems with extension systems limit technological diffusion [12]. For example, misperceptions about profitability, labour demands, or risk—combined with mistrust of external advice—can hinder adoption of yield-enhancing practices [16]. Farmers can be overwhelmed by competing recommendations or sceptical of unfamiliar technologies [17,18]. Moreover, top-down efforts may sideline already marginalized communities like women or indigenous groups by downplaying the salience of their needs or characterizing their existing ‘extensive’ practices as backwards, leading to greater distrust and resistance [19,20].

### 2.2. A sociocultural perspective

Recent advances in behavioural and social theory demonstrate that agricultural decision-making is deeply **enculturated***,* influenced by social and cultural processes*,* and ‘**enearthed**’*,* embedded within biophysical systems [21,22]. Farmers’ existing practices are shaped by identity, values, family tradition, experience and sense of place, among other factors, not solely by profit maximization, which is why they rarely follow the logics of intensification. What constitutes ‘sustainability’ or ‘success’ in farming varies across contexts, and practices that align with external definitions of efficiency may conflict with local notions of stewardship, religious ethics or desired ways of life [23]. Both risks and rewards are also often generated at the community level. For example, farmers may be unwilling to invest in SI if that investment can be wiped out through escaped fires from neighbours’ farms or a lack of water from a neighbour's excess withdrawals [24].

### 2.3. A structural perspective

Structural frameworks such as the multi-level perspective [25] and food regimes [26] focus on understanding how broader political and socio-economic conditions enable and constrain farmers' choices and shape their preferences. From these perspectives, SI can be considered a type of niche activity. Its scaling is affected by processes at the *landscape* and *regime* levels [25,27]. Food regimes are the dominant configuration of agrifood production systems, supply chains, policies, markets and scientific paradigms that constrain change, causing many SI niches to remain ‘trapped’ (unable to scale) [26,28]. At the landscape level, slow-moving social and environmental forces exert pressure on agricultural systems. These include global political-economic systems (e.g. capitalism and colonialism) and socio-technical trends (e.g. globalization, financializaton, demographic change and climate change) [29]. Landscape factors can also include the **socio-technical imaginaries** that constrain or open up future options [30], including competing visions of land sparing and land sharing for food and nature synergies and techno-optimist beliefs in individual innovations as a panacea for food and nature challenges.

In lower- to middle-income country contexts, food regimes have been heavily shaped by international financial actors and post-colonial development trajectories. Historically, colonial investments in agriculture prioritized export-oriented, low-value production [31,32]. Following debt crises in the 1970s, structural adjustment programmes enforced by international financial institutions forced the restructuring of national economies through reductions in public spending and trade barriers [26]. Restructuring efforts also dramatically cut public investment in rural services and deepened a focus on export commodities to service debts [26,33,34].

The further globalization of food markets following the reductions in trade barriers in the 1990s has resulted in an increase in the length of value chains, that is, an increase in the number of actors and physical distance between producers and consumers [35,36]. This, in turn, has contributed to the concentration of market power in the middle of food supply chains, with fewer companies controlling a large proportion of food that gets exported. Mid-tier actors (i.e. food processors and traders) in these chains occupy a powerful role in shaping prices, technology choices and information, which dramatically constrains farmers’ incentives and agency to adopt different practices [37,38]. Mid-tier actors are also key players in the development of sustainability and quality standards that provide both a challenge and an opportunity for small farmers in the context of SI [39]. They create additional compliance costs that could detract from SI investments [40,41], but could also promote SI if the standards focus on good agricultural practices and closing yield gaps [29,37].

In many low-income regions, entrenched poverty can be considered as another key factor limiting SI. Additionally, in many regions throughout the world, farm sizes—owing to multi-generational fragmentation—may simply be too small to repay the investment costs if those costs were to be incurred through borrowing, and even if yield gaps are closed, the household will still be unable to earn a living income or escape poverty [42,43].

Although landscape factors are seen as external to the food regime, certain changes in landscape factors can destabilize the dominant regime and create ‘intensification windows’—a shift in conditions that create an opening for SI [44]. For example, changes in input markets, product prices and yields from abrupt weather, conflict and demand shocks can all create new windows for SI [45].

### 2.4. Putting it all together: components of yield gaps

Putting these theoretical barriers together at multiple scales can help us to understand why actual yields fall short of technological potential. Figure 1 illustrates this logic (see caption for an explanation of each step), helping to connect dimensions of the conceptual framework to different components of the overall yield gap on a given farm within a particular community. As the figure highlights, actual yields, i.e. what farmers are really obtaining in their field, are pushed down by barriers at multiple scales. The community- and farm-level barriers outlined above affect the community potential yields, and regime-level barriers shape real-world conditions and total attainable yield, as well as barriers at community and farm levels. The stylistic error bars within each bar are a conceptual tool to represent the heterogeneity in yield gaps across different communities and households within a given region, which are, in turn, linked to the diverse constraints and motivations experienced by these different groups and individuals. This multi-level approach can complement other transversal frameworks, such as [12].

**Figure 1.**
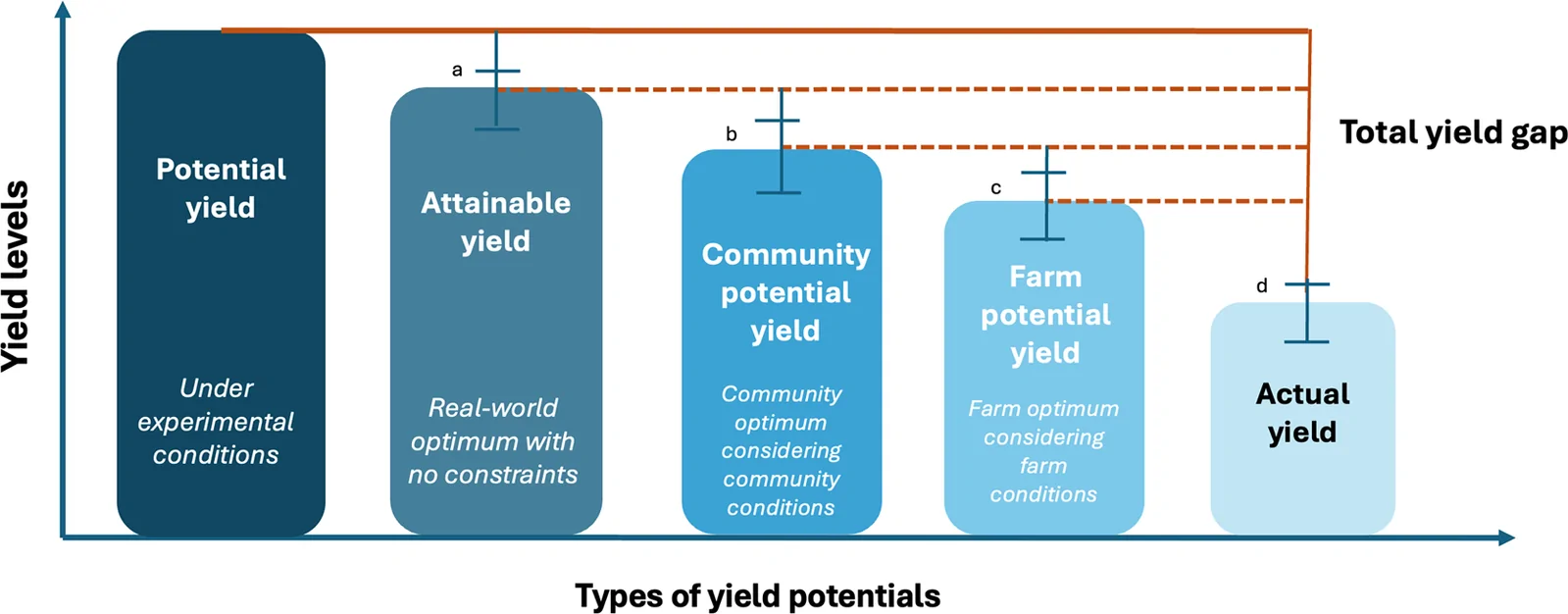
Different conceptual groups of yield potentials to guide understanding of the varied drivers of yield gaps. Notes: (a) is the difference between the experimental maximum yield and real-world conditions at the optimal technology, for those farmers and communities that have sufficient access, awareness and capacity; (b) is the difference between the real-world optimum and what communities could achieve with all collective action problems solved, given their regional constraints; (c) is the difference between community-constrained optimum and the farm-level optimum given farm characteristics, like size, soil quality, labour and physical capital. The farm optimum includes considerations of profitability, which may rationally entail lower input use and yields; and (d) is the difference between the farm-level optimum and what different farmers achieve given a lack of awareness, access and resources. Error bars note the high deal of heterogeneity within each conceptual group, e.g. different farmers within a community, communities with different constraints.

## 3. Conditions for sustainable intensification

The above collection of theories helps explain why even potentially profitable and sustainable SI practices do not scale within certain agricultural regimes, and indeed, why yield gaps are so prevalent in lower- to middle-income country contexts. While daunting, these conditions create an **intensification opportunity**, i.e. a substantial gap between what a place is producing in terms of quantity or value and what it could produce given excess demand for that product that is unlikely to be met in other ways (e.g. lowering food loss/waste or shifting diets). *But what conditions are needed to seize this opportunity?*

First, there must be a **feasible innovation bundle** available to close the yield gap, i.e. different cultivars/genetic stock or management practices, and other bundles of institutional, policy or techno - logical innovations exist that can feasibly close the gap [46]. It is important that this bundle of technologies will not be undercut by climate hazards, or else the related SI could promote maladaptation [47], particularly sunk costs in pathways that will not produce the greatest food–nature synergies. Next, conditions must align at the (i) farm level, (ii) community level and (iii) broader regime to support SI.

At the farm level, capacity, motivations and security all influence whether farmers will undertake SI. If agents have sufficient financial, technical, knowledge and infrastructure capacity, they will be more likely to adopt innovations and absorb risks inherent to adoption. Agents have diverse motivations, which can be financial, labour-related or sociocultural, among other factors. If the SI increases profitability, reduces risk, saves labour, reduces managerial complexity and/or aligns with heritage, experience, social norms and dietary preferences, it will be more likely to be adopted. Agents will be more likely to invest in SI if they have certainty they can benefit from the longer-run pay-offs of their investments. Risk of not achieving these benefits is lower when farmers have sufficient resource tenure security, longer demographic horizons (e.g. more years of life remaining or clear farm succession) and greater physical safety (an absence of local violence and conflict).

At the community level, local institutional and infrastructural factors such as reputation, finance, marketing and collective action are all critical factors influencing SI. Economies of scale (agglomeration) and value chain improvements all create new opportunities for farmers [50]. An individual farmer's decision to intensify will depend on whether others in the community are undertaking behaviours that affect their costs or benefits of adopting the SI. The negative and positive externalities from individual behaviours make many SI decisions a collective action challenge. Peer effects also matter to shortcut decision-making burdens, and some farmers only act when they see other lead farmers adopting technologies [48,49]. Similarly, shared group norms and values can influence SI. Intensification is more likely where the SI results in improved social status (e.g. if advanced technologies, productive fields or fat cows are associated with prestige). Finally, it is essential that there is reliable and affordable access to finance, inputs and access to remunerative and stable marketing pathways for surplus output (e.g. developed supply chains and public procurement programmes).

At the regime level, input and product markets and broader politics and power dynamics all play an important role in whether SI occurs. When land becomes scarcer, but labour and capital remain the same or increase, it can help ‘push’ the intensification from the bottom by improving the cost-effectiveness of investing labour and capital into productivity increases on the existing land instead of focusing on expanding the production area [50,51]; for example, if land is removed from production owing to land gazetting or other policy change. Similarly, when the market value of the product increases owing to demand shifts, it can ‘pull’ intensification from the top by improving the return on investments like new genetic material or fertilizers [52], but this depends on whether land expansion remains constrained. Population growth without increases in land protection (and scarcity) can just promote further land expansion. Finally, SI is more likely when it results in at least some benefits to powerful actors in the system (e.g. through sales and marketing channels or tax revenues or security and sovereignty narratives) [46].

The conditions supporting SI are summarized in figure 2. In the sections that follow, this conceptual framework is applied to the two case studies to examine how SI unfolds in practice within biodiversity-rich regions.

**Figure 2.**
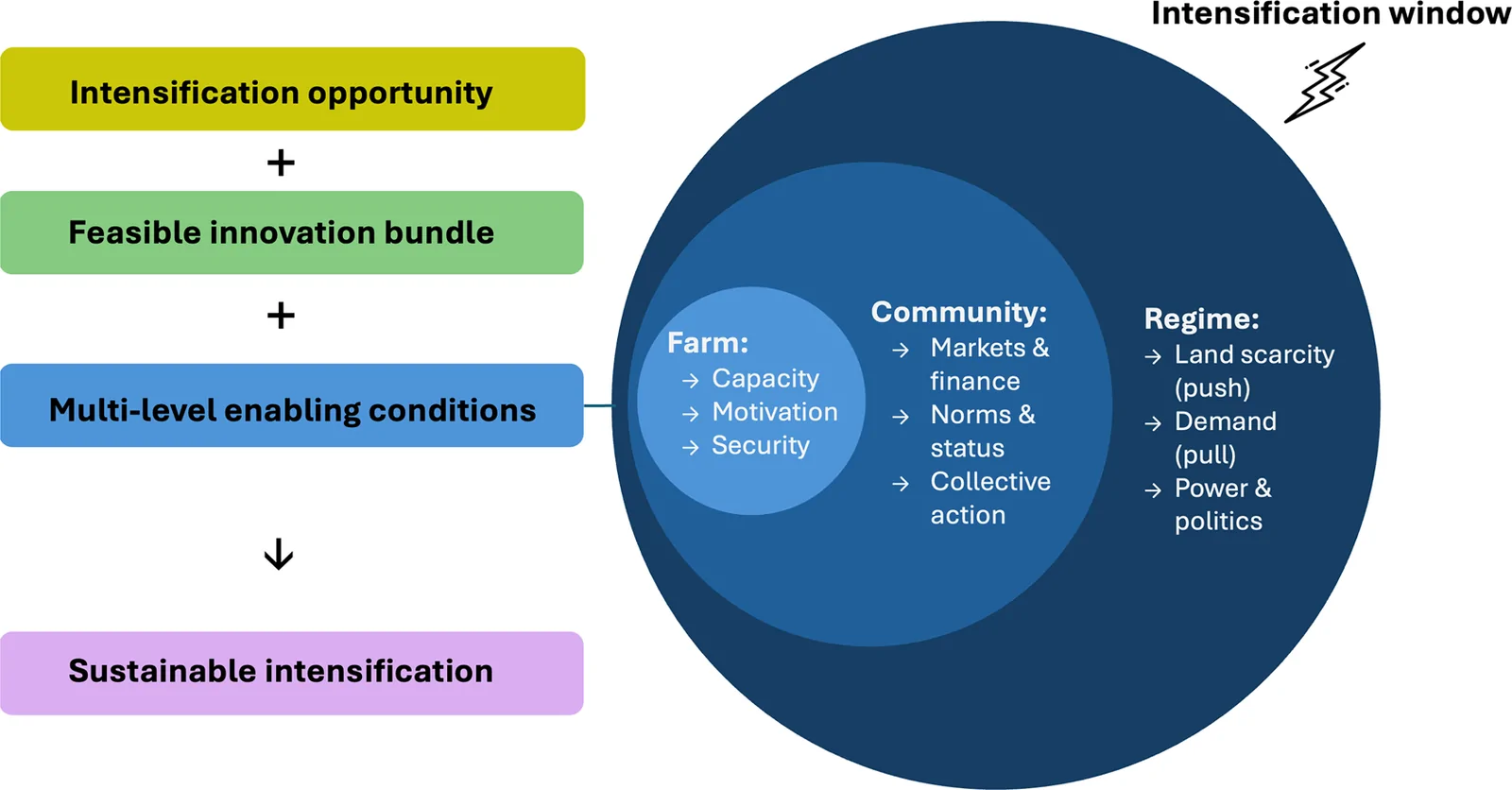
Conceptual model of how sustainable intensification can occur.

## 4. Beef cattle production in the Brazilian Amazon and Cerrado

### 4.1. The intensification opportunity

The Brazilian Amazon and Cerrado harbour some of the greatest biodiversity in the world. Yet both places have rates of native vegetation loss that are among the highest in the world, almost entirely owing to agricultural expansion. Chief among the agricultural drivers of deforestation is cattle ranching, which occupies 80% of the cleared areas in the Amazon [53]. Cattle pastures are also the largest single land use in Brazil, occupying 154 million hectares as of 2024 [53]. Including both its land cover impacts and the methane emissions of live cattle, beef cattle production is the single largest source of emissions in Brazil, accounting for 50% of the gross emissions [54].

Pasture areas are dominated by extensive practices (i.e. continuously grazed with almost no soil correctives or new seeds). Extensive practices degrade the soil, cause cattle to gain weight slowly and even lose weight during the dry season, leaving productivity at only 30% of its potential [55]. Fires are often used to manage vegetation regrowth on cleared areas [56]. These fires often escape from the areas where they were started, cause numerous economic and health losses to local communities and destroy agricultural infrastructure [57].

### 4.2. Feasible bundle of innovations

Reversing degradation and transitioning to more sustainable and productive practices is not only entirely possible, but could significantly improve incomes for the farmers adopting these practices [58]. Integrated crop–livestock systems (ICLS), improved genetic stock and cattle supplementation have all been shown to improve productivity and ranching profits while providing more diversified income [59]. Indeed, prior to 2006, significant gains in cattle productivity were made by advances in the forage material, including improved mixed grass-legume pastures [60]. Without such improvements in yields, more than three times more pasture would have been needed to obtain the same levels of production [61].^1^

SI of cattle ranching has been heavily promoted by the Brazilian government's low-carbon agriculture and deforestation prevention policies for over two decades. The government provides subsidized loans for pasture reformation and integrated systems, and has improved restrictions on native vegetation clearing, which reduces the attractiveness of further clearing in many areas [16,50,62]. Despite these efforts, many farms do not adopt the available technologies, and ongoing extensive pasture expansion continues to deflate the average performance of the sector [61,63].

### 4.3. Barriers to intensification

#### 4.3.1. Farm level

*Capacity:* despite the objectively large size of cattle ranches, many farmers are still considered ′family farmer′ (i.e. smallholders) under Brazilian law owing to their relatively smaller sizes in the Brazilian context and their high reliance on family labour. These family farmers comprise 78% of the cattle farms in the Amazon and Cerrado and are heavily capital constrained. Consequently, the high upfront costs of many SI approaches are beyond their means without access to credit [64,65]. Yet, for many farmers, the larger and longer-term loans needed for SI investments are unavailable owing to a lack of formal land tenure [16,66]. *Motivations:* the preferences of ranchers also tend to be biased towards low management intensity (i.e. letting the cattle graze continuously in extensive systems) and a strong aversion to taking on debt [16,62]. The low management intensity means that extensive ranching has the additional benefit of lower hired labour requirements and associated costs and risks. Meanwhile, for wealthier farmers, the high sociocultural prestige and asset accumulation implications of occupying large land areas (even if they are not profitable) further entrenches extensive practices [66,67].

#### 4.3.2. Community-level barriers

*Risk:* the transition to more intensive ranching involves shifting away from fire use to manage vegetation regrowth. But in the context of neighbours’ use of fires and resulting escaped fires, no individual farmer has the incentive to invest in fire-free approaches [24]. This makes reformation a collective action problem in addition to an individual behaviour challenge. *Finance and market options:* the Brazilian Government provides a wide range of subsidized credit lines targeted at both family farmers and agribusiness [68]. Yet, the offerings provided by the government tend to be inadequate for transforming farming practices owing to inadequate funding levels, high interest rates and short-term payback periods [16,62]. Ultimately, much of the credit ends up being allocated to large-scale farmers who have separate lines of funding and significantly better access to private extension [68].

#### 4.3.3. Regime barriers

*Factor abundance:* incentives continue to favour expansion since forested land is artificially cheap owing to a prevailing economic system that does not sufficiently value forest conservation and overvalues cattle production (bynot internalizing the massive environmental harms of the sector). These outcomes are in turn linked to a longer political economy trajectory within Brazil focused heavily on cattle ranching as a mechanism to colonize and settle land, drive out indigenous groups and safeguard the Amazon region from foreign interests [31]. Land speculation itself is a major economic motive to maintain extensive practices, particularly where land is cheap but rapidly increasing owing to the advancing soy frontier [69]. *Adverse product markets:* while beef prices have increased substantially in recent years, profits remain low for producers, and a lack of high-value marketing options still constrains SI adoption. Beef demand in Brazil is dominated by domestic markets, constraining the reach of international sustainability standards to promote SI. *Food regime politics:* farmers seeking higher value markets face limited negotiating power since purchases are highly concentrated among three major federally inspected slaughterhouses [70]. These slaughterhouses wield significant influence in Brazilian politics, including close links to both major parties. State and local slaughterhouses are available, but typically provide undesirable marketing terms, reducing the prices farmers receive owing to unfavourable carcass weight measurements and delayed payment.

### 4.4. Where does intensification occur and why?

While intensification remains low across many areas of the Amazon and Cerrado, there are notable exceptions. Firstly, there are numerous areas where soy production and other more valuable crops (e.g. maize and cotton) have replaced extensive pastures. From 2000 to 2023, 35 million hectares of pasture were replaced with crops, and 81% of the pastures that transitioned were those of low to medium quality [53]. This intensification was driven, in part, by the strengthening of deforestation regulations, but also by increasing demand for crops like soy [50]. Both public conservation regulations and zero-deforestation supply chain policies have helped to reduce the relative benefits of expanding land area in the Amazon rather than intensifying by increasing the de facto price of forested land. Regulations in the Cerrado are less stringent than in the Amazon, but have been strengthened through the Plan for Control and Prevention of Deforestation in the Cerrado, but soy farmers are also buying or renting existing pasturelands, further displacing some of the existing pasture area. While most of the soy expansion in the Cerrado was driven by increases in demand in China, some of this expansion of soy in the Cerrado is linked to leakage from zero-deforestation policies for soy in the Amazon [71].

The European Deforestation Regulation (EUDR), a demand-side regulation which requires importers to ensure that the supply of certain high deforestation-risk food products, including beef, does not come from land deforested after 2020 presents an additional opportunity to signal increasing demand for deforestation-free production and support restrictions on further pasture expansion into forests in both the Amazon and Cerrado; however, it currently excludes other woodlands, limiting its potential effectiveness in the Cerrado. The United Kingdom Illegal Deforestation Regulation (UK-IDR), which requires that importers ensure their supply did not come from lands that were illegally cleared after 2020, complements the efforts of the EUDR.

Where cattle remain, there has been an uptick in wealthier farmers adopting more intensive systems. These include semi-confinement and improved cattle supplementation, as well as rotational grazing and integrated crop–livestock systems. Intensification is higher near established cropping areas and surrounding experimental units and demonstration farms [72]. Yet these efforts are not adopted by most cattle ranchers in the Amazon, and do not change the fundamentals of cattle as a speculative activity. However, shifts in demand for cattle slaughtered at a younger age and with higher fat content have supported some of this intensification [73].

## 5. Cocoa production in the Upper Guinean Forests of West Africa

### 5.1. The intensification opportunity

The UGF are a global biodiversity priority area, characterized by high levels of endemism and vascular plant and mammal diversity [74]. Yet only 15% of the forests remain owing to pressure from agricultural expansion, namely cocoa and staple food crops [75]. The two major hotspots of forest loss for cocoa are Côte d'Ivoire and Ghana, where cocoa accounts for more than 45% of total deforestation and degradation between 2000 and 2019 (3.2 million ha) [76,77].

Yield gaps on cocoa farms are sizeable. In some places, cocoa plots are only achieving 27% of their maximum attainable yields [78]. The prevailing practices include old cocoa trees, low input use and fallowing after areas become too diseased or degraded. Low-productivity cocoa can be a major threat to the remaining biodiversity. As yields and soil fertility decline on existing farms, farmers seek new forests to produce food and expand their cocoa plots [77,79,80]. However, existing low-intensity cocoa systems still provide numerous other food products as part of more complex mixed agriculture or agroforest systems. Understanding of the total factor productivity of these areas is limited.

### 5.2. Feasible bundle of innovations

There are numerous ways to improve yields in cocoa areas, including both higher-cost approaches that are unlikely to be viable, as well as lower-cost options. It is estimated that cocoa yields in West Africa could be more than doubled with basic agronomic training and practices, including the use of fertilizers and disease control [81,82]. For example, field trials in four West African countries indicate that more accurate targeting of fertilizers could improve upon both current practices and no-fertilizer best practices by 34.5 and 33.8%, respectively [83]. Another study estimates that using higher planting densities and black rot fungicides alone could reduce 42% of the yield gap [84]. Improvements in yields have also been shown to reduce school absences and improve food security [81]. An economic modelling analysis suggests that increasing farm cocoa yield from a current average of close to 600 kg ha^–1^ in the cocoa farms studied to 1500 kg ha^–1^ with best practices could help reduce the percentage of cocoa households with incomes below the living income benchmark from 82 to 20% in Côte d'Ivoire and from 73 to 13% in Ghana [85].

Despite ample opportunities to improve yields, productivity is also severely threatened by increasing dry season temperatures. If the maximum temperature of the warmest month increases over the 2015 average by 1.5°C by 2050 (a likely scenario), then the areas highly suitable for cocoa would reduce by 50% in Ghana and Côte d'Ivoire [86]. To combat climate stress and improve soil fertility, many cocoa sustainability interventions focus heavily on the use of agroforestry (intercropping taller tree species over cocoa) to provide shade [87]. Though some studies show that yields may start to decline after 30% shade cover [88], other studies estimate that cocoa yields can double when shade is increased from 1 to 24% shade cover [89]. It is estimated that agroforestry can reduce the microclimate by 0.32°C for every 1°C temperature increase [90]. It has also been suggested that agroforestry can help improve fertilizer efficiency in cocoa systems, but more research is needed [91].

### 5.3. Barriers to intensification

#### 5.3.1. Farm level

*Capacity:* cocoa farmers in the UGF are very poor. Van Vliet *et al.* [85] estimated that 30–58% of households in Côte d'Ivoire and Ghana earn a gross income below the World Bank extreme poverty line, while 73–90% do not earn a living income. More than 90% of the cocoa supply comes from smallholders [92]. While most native farmers are highly knowledgeable about cocoa production owing to its long-term presence in the region, they are not always knowledgeable about the technical aspects of improving cocoa yields, especially since historical practices used more extensive fallowing and agroforestry practices, relying on fertility boosts from newly cleared vegetation [82,93]. *Motivation:* high poverty levels and dependence on rural activities for income make improved financial returns a key motivation for SI. Yet, food security is another critical motivation. While many cocoa farmers are food secure on average, dietary diversity and food sufficiency drop substantially during the lean cocoa season from reduced income. Maintaining diverse food crops on farm is thus another factor driving lower cocoa yields per hectare. When SI provides additional income security, e.g. from intercropping marketable food or timber species while also boosting cocoa yields, it is more likely to be adopted [94]. *Risk:* some SI approaches (e.g. planting new cocoa trees and agroforestry) require high upfront investments without short-term pay-offs, making them out of reach for most farmers [95–98]. For example, most perennial food trees, including new cocoa trees, usually have a 3+ year lag before they start producing. Furthermore, even more immediate improvements like fertilizer use are still costly and pose risks if the crop is lost owing to disease or other hazards. These high upfront costs, alongside a lack of land and tree tenure, increase risk and limit farmers’ willingness to invest in soil improvements or plant beneficial trees [94,99–101]. The lack of land tenure also affects credit access, which is already scarce.

#### 5.3.2. Community level

*Capital and market options:* a lack of government investment in technical support, infrastructure and loan programmes means that smallholders face heavily constrained access to agronomic knowledge as well as quality fertilizers, fungicides or improved planting material. *Risks:* while improved yields have a direct income incentive, there is a high degree of risk of lost income in investments owing to disease outbreaks, in particular swollen shoot and black pod diseases [78,81]. These disease outbreaks are community dependent, as they spread from tree to tree. Conversely, reducing both disease and climate risks is a collective action problem, requiring proper cocoa tree management and shade cover at the community level.

#### 5.3.3. Food regime

*Factor scarcity:* climate change has reduced the suitability of traditional cocoa-growing zones, particularly in lowland areas of the UGF. However, numerous additional forest and fallow areas remain accessible for cocoa expansion, limiting pressure to intensify production. This is especially true for newer cocoa frontiers: Nigeria, Liberia and Sierra Leone [102]. *Risks:* rising temperatures, altered rainfall patterns, and increased frequency of droughts directly reduce cocoa productivity by increasing evapotranspiration stress, and pest and disease pressure. Climate change also shortens the viable lifespan of cocoa trees. These pressures increase yield risk and uncertainty, discouraging long-term investments in rehabilitation or intensification [80,103]. *Product markets:* while global cocoa prices were very high in 2024–2026, they are also highly volatile. In Ghana and Côte d'Ivoire, the cocoa marketing board heavily regulates prices and exports. This has the advantage of maintaining a price floor for farmers; however, the prices are still below the average global price, and the fixed pricing disconnects farm incomes from world market improvements (including recent price surges). The overall low prices and the inability to see the benefits of rises in prices reduce incentives to intensify production in existing regions [87,90]. *Politics:* cocoa supply chains are dominated by a small number of multinational traders and manufacturers, limiting farmers’ bargaining power*.* The national market and institutional system have been shaped by post-colonial political–economic legacies that leave both farmers and nations ill-equipped to grow agricultural productivity. Structural adjustment policies in the 1980s–1990s reduced public support for extension, research and rural finance, weakening long-term yield growth. And the low economic development in turn constrains growth of the agribusiness sector. To date, private sector efforts have not been disruptive enough to make a dent in existing cocoa trajectories [87,90]. Instead, they accommodate existing power dynamics that contribute to the ongoing challenges.

### 5.4. Where does intensification occur and why?

The doubling of cocoa yields in Ghana between 2003 and 2019 [104] serves as a powerful example of how intensification windows can be provided by international landscape changes, including carbon markets and international development finance. Widening access to inputs and extension, development of improved varieties and adoption of climate-smart practices, including agroforestry, have supported cocoa yield improvements. These productivity-enhancing efforts were financed by large increases in funding from the World Bank and the UN Reducing Emissions from Deforestation and Degradation (REDD+) mechanism, which have facilitated the Ghana Forestry Commission's efforts to improve cocoa practices [105]. REDD+ is complemented by a wide range of public and private sector efforts to target cocoa productivity in the country [87,90]. Most recently, in 2026, the World Bank pledged another 75 million USD to support cocoa intensification, providing a new powerful window for SI [106]. Zero-deforestation supply chain policies, the EUDR and the UK-IDR also apply to cocoa. However, the implementation of these important demand-side levers to stimulate intensification by increasing the costs of deforestation has not been as stringently implemented as in the case of beef cattle production [107]. However, some firms do include some yield-enhancement interventions within their overall zero-deforestation strategies [90]. The impacts of these interventions on cocoa yields have not yet been measured.

## 6. Discussion of the case studies

There is a similar opportunity to triple yields through the adoption of SI in both the Brazilian beef cattle and UGF cocoa systems. Furthermore, both places face remarkably similar barriers to SI at the farm-to-regime scales (figure 3). In both tropical forest regions, historical low productivity has exacerbated low incomes, leading to lower capacity to invest in SI, creating a lock-in for farmers with low-yielding systems. At the farm level, most farmers are smallholders (though cattle farms are significantly larger) who are capital constrained and lack tenure security, which is problematic given the high upfront costs of SI investments. At the community level, farmers in both places face fire and disease risks from SI in the context of their neighbours’ behaviours. These conditions make SI both unaffordable and risky. At the regime scale, both cases share high forest abundance (making land expansion relatively cheap), low market prices for the agricultural product (beef and cocoa) and limited farmer power relative to multinational corporate buyers. In both places, a growing push from international biodiversity and climate conventions, and associated international climate and development finance, are creating new windows for intensification, but their scale is still insufficient to drive widespread change. Increased heat and water stress from climate change is a broader landscape factor that is already inhibiting cocoa productivity and will exacerbate dry season challenges for cattle.

**Figure 3.**
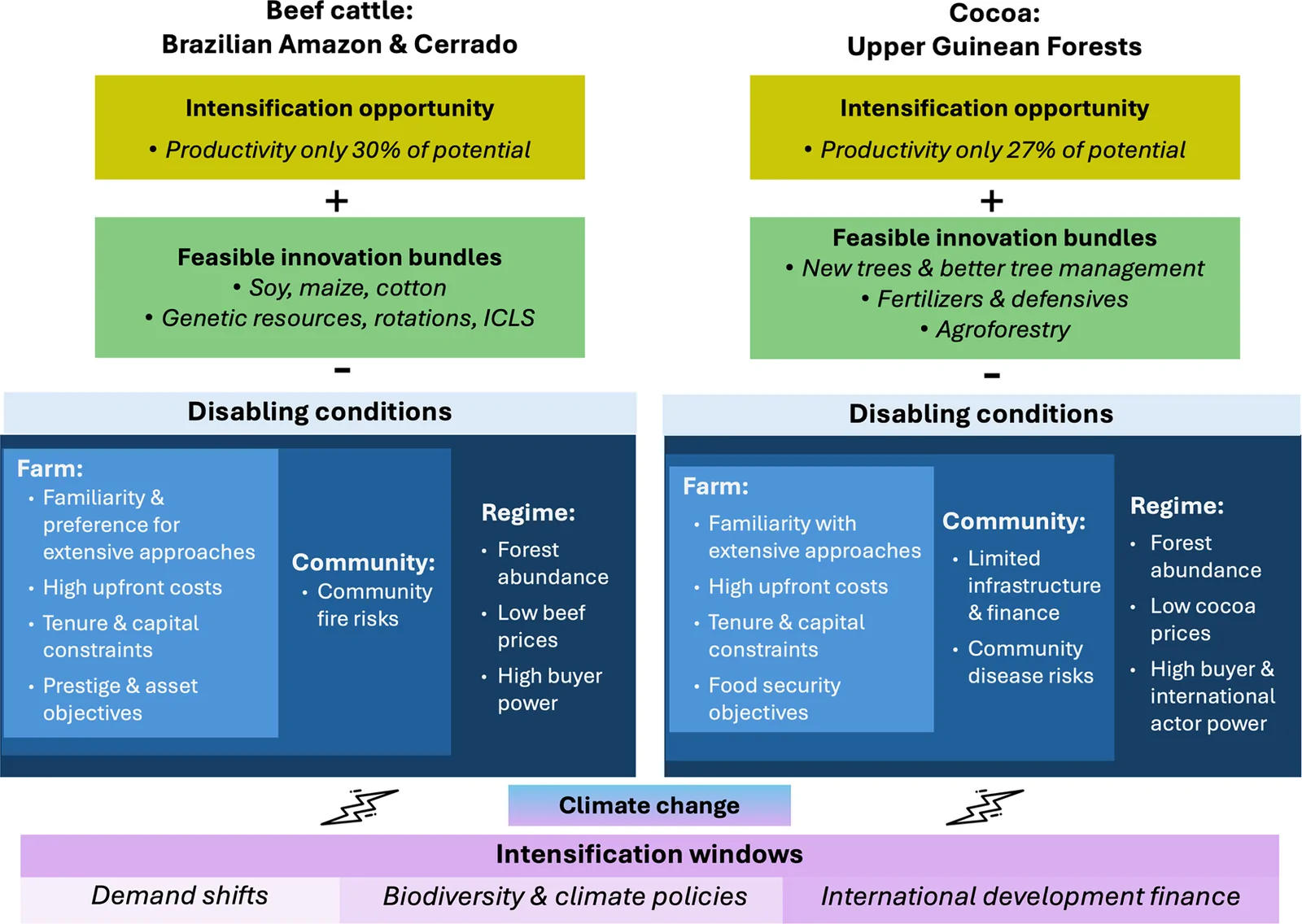
Summary of intensification opportunities and barriers across the two cases.

The main difference between the cases is that in Brazil the government has invested a large amount of money in both improving conservation policy and improving agricultural practices, leading to relatively large and effective environmental enforcement and rural credit systems. Yet Brazil still must contend with huge amounts of undesignated and unregulated forest lands and cultural lock-ins owing to historical cattle frontier expansion narratives [69]. By contrast, countries in the UGF must rely on external development and climate finance options with narrower interests, as well as stronger adverse colonial legacies that have weakened traditional tribal institutions [102]. Niche sustainability efforts focusing on SI have yet to make a dent in closing yield gaps amid this greater regime-level counter pressure.

These cases underscore how closing yield gaps is not a purely technical challenge but a deeply wicked problem. Agricultural systems are shaped by interacting social, economic, cultural and biophysical processes that create path dependencies and lock-ins. Behaviours that appear optimal from an agronomic perspective may be misaligned with farmers’ objectives and capacities, especially in the context of risk. Climate change offers a challenge by further reducing yields, but also potentially a window by underscoring the urgency of directing greater attention to low yields in our fragile and volatile global food system. New import policies in Europe and the UK requiring zero-deforestation and deforestation legality, respectively, also present an opportunity to shift demand in ways that could encourage intensification.

## 7. Risks of sustainable intensification and safeguards

Increased inputs, new cultivars and longer-term investments in SI can create economic and environmental challenges for farmers without sufficient guard-rails. If investments fail to achieve their expected returns, farmers can go into debt, resulting in an exit from agriculture and livelihood harms, including suicide. Farmers must have greater access to insurance and secure tenure to avoid catastrophic losses from higher investments in crop yields. For example, there are calls to shift money from conventional crop insurance programmes in the USA towards enabling new entry of sustainable farming options [108].

Additionally, expanding SI in frontier areas runs a high risk of incentivizing further clearing if it increases income (i.e. a rebound effect). To avoid additional clearing, it is essential to strengthen conservation policies in areas where SI is pursued. If local protections are lacking, then at a minimum there must be strong international governance efforts in place to control deforestation and support land sparing. For example, Brazil has coupled investments in loans for low-carbon agriculture practices with conservation restrictions, which has helped reduce some deforestation and promote some SI of the cattle sector (though challenges with the credit programmes and deforestation enforcement remain) [62].

## 8. Conclusion

Sustainably closing yield gaps remains one of the greatest challenges to improving global food security and conservation. This article has provided a theoretical perspective on why yield gaps persist despite the urgent need to improve the productivity and value of agricultural areas for social and environmental benefits. We outline factors at the farm, community and regime levels that constrain adoption of SI and explore these through two case studies in expanding commodity frontiers in the tropics:Brazilian Amazon and Cerrado cattle and UGF cocoa.

The cases show how multi-level factors significantly constrain SI despite (i) ample opportunities for intensification (given the size of yield gaps and volume of excess demand) and (ii) numerous feasible bundles of innovation that can achieve this SI. The disabling conditions are remarkably similar across the two cases, pointing to their potential broader significance in similar settings. The major regime barrier in both regions is the low price of forest land (and lack of internalization of deforestation externalities), exacerbated by flawed development logics, which enables artificially low costs to extensification over intensification. The cases presented here are therefore not fully generalizable to places where the potential for expansion is low and whether other regime barriers to SI dominate. Nevertheless, the cases remain illustrative to explore some of the SI barriers that dominate in more land-abundant regions—precisely the places where remaining biodiversity is most threatened.

The good news is that ambitious economic and institutional reforms, when paired with smart conservation policies, can unlock benefits for both food security and biodiversity conservation. Ambitious and transformative approaches must seek to adjust the broader systemic economic conditions and community risks that inhibit change [23]. This necessitates a greater investment in a community-by-community ‘big push’ approach so as not to dilute resources. These investments must aim to be ‘disruptive’ enough in their scope to change all behaviour in a community and shift social norms to overcome collective action challenges. Efforts should include entire innovation bundles and related capacity building (tenure security and supply chains) to overcome regime lock-ins and align incentives and governance. The cultural aspects of SI challenges underscore the need for participatory, adaptive and localized diagnoses of SI challenges and solutions that resonate with farmer values, preferences and constraints, rather than pushing technologies that seem theoretically optimal under simplified behavioural assumptions. For example, the oil palm sector in Indonesia has developed a Fire-Free Village programme to tackle yield-undermining fire risks at the community level [109], and companies are investing in longer-term relationships with farmers, including comprehensive support programmes for good agricultural practices and financial literacy to enhance yields [110].

To ensure broader sectoral support and buy-in, those developing SI innovation bundles, including both the technologies and associated government policies, should build in wins for both farmers and more powerful supply chains [111]. Coupling SI technologies with investments in rural infrastructure, land tenure and extension has proven successful in some countries [46]. These gains are especially likely when international environmental and development policies, financial flows and demand shifts create favourable windows for change. In such moments, e.g. price spikes and famines that catalyse public attention, coordinated action can turn competing priorities into a shared pathway towards more resilient and sustainable outcomes. Actors working in the SI space should be ready to take advantage of ‘intensification windows’ provided by local or broader landscape changes.

## Data Availability

This article has no additional data.

## Footnote

^1^
This is not the same as land sparing as it does not consider the counterfactual of what would have occurred without these yield increases.

## Glossary

Yield gaps: the difference between the agronomically attainable productivity of an agricultural land use (crop/livestock) and what is achieved in farmers’ fields.
Sustainable intensification: increasing agricultural output or value from the existing land while maintaining or enhancing environmental integrity and resilience.
Extensive production: farming practices that use few inputs and extend over larger areas to produce the same amount of food as could be produced on a smaller amount of land with more inputs.
Enculturated and enearthed behaviours: behaviours that are influenced by local social and cultural processes and biophysical systems.
Food regime: configuration of production systems, supply chains, policies, markets and scientific paradigms that characterize the food system.
Food landscape: slow-moving social and environmental factors external to the food regime that can shift or destabilize the food regime.
Food niche: agricultural or food practices, technologies or organizational forms that are not widespread, including novel/emerging agricultural or food practices, technologies or organisational forms and once dominant, but now marginalised land traditions.
Scientific imaginaries: shared/collective visions or ideas about how science and technology shape the world, especially the future, including what is possible, desirable or risky.
Intensification opportunity: a substantial gap between what a place is producing in terms of quantity or value and what it could produce given excess demand for that product that is unlikely to be met in other ways.
Intensification window: an opportunity that arises to overcome barriers to SI when the food regime or landscape changes.
